# Case Report: Multimodal clinical and neurophysiological effects of cranial electrotherapy stimulation in a patient with chronic stress and sleep disturbance

**DOI:** 10.3389/fpsyt.2026.1808277

**Published:** 2026-06-03

**Authors:** Ellie Mitsi, Sergey Kondratev, Petros Kattou, Stavria Georgiou

**Affiliations:** Research Department, SOZO Brain Center, Nicosia, Cyprus

**Keywords:** CES, EEG, anxiety, sleep disturbance, neuromodulation, NIBS, case report

## Abstract

**Introduction:**

Cranial Electrotherapy Stimulation (CES) is a non-invasive brain stimulation (NIBS) technique used for anxiety and sleep disorders; however, case-level evidence linking clinical improvement with objective neurophysiological change remains limited. This case presents a multimodal examination of the therapeutic and neurophysiological effects of CES in a patient with sleep disturbances and chronic stress.

**Methods:**

A 51-year-old woman with chronic stress, anxiety, and persistent sleep disturbance following thyroid and parathyroid surgery, refractory to pharmacological treatment, underwent a 16-week CES intervention. Outcomes were assessed at baseline and follow-up using EQ-5D-5L, DASS-21, GAD-7, PSQI, and resting-state Electroencephalography (EEG).

**Results:**

Anxiety, stress, and depressive symptoms improved to within normal ranges, alongside significant improvements in mental health-related quality of life. EEG demonstrated increased posterior alpha activity and improved frontal symmetry. Sleep quality showed modest improvement.

**Conclusion:**

This case suggests that CES may be a safe adjunctive intervention associated with symptomatic improvement and changes in neurophysiological patterns in stress-related conditions, supporting further controlled investigation.

## 1 Introduction

Chronic stress and associated sleep problems contribute to a major public health concern, leading to a broad spectrum of mental and physical health conditions (1). Conditions such as generalized anxiety disorder (GAD) and chronic sleep disturbances are highly prevalent and can severely impair an individual’s quality of life and daily functioning (2). Although pharmaceutical and psychotherapy treatments are frequently used, they are not always successful or well-tolerated, requiring the use of complementary and alternative therapies.

CES is a non-invasive neuromodulation intervention that delivers low-intensity alternative electrical current to the brain via electrodes placed bilaterally on the head, most commonly at the earlobes or mastoid regions (3, 4). CES is approved by the U.S Food and Drug Administration (FDA) for the treatment of anxiety, depression, and insomnia (5), while it has been investigated for several decades as a therapeutic intervention for neuropsychiatric and stress-related conditions, including anxiety, depression, insomnia, and chronic pain syndromes (6, 7), owing to it favorable safety and ease of administration. Early and subsequent clinical trials, systematic reviews, and meta-analyses have reported modest but significant reductions in anxiety and depressive symptoms with CES compared to sham controls, with overall moderate effect sizes and high tolerability across diverse populations (8, 9). The precise mechanisms underlying the effects of CES are still under investigation. However, studies suggest that the electrical currents, though of low intensity, can reach cortical and subcortical brain structures (3). Neuroimaging and electrophysiological studies have observed that CES can modulate brain activity, for instance by increasing alpha band EEG activity and altering connectivity within the default mode network. Also, it has been hypothesized that CES may exert its therapeutic effects by modulating brain stem and limbic regions, thereby increasing parasympathetic tone in autonomic nervous system, possibly through stimulation of vagal nerve afferents (3).

The present case report aims to address this gap by presenting a multimodal investigation into the clinical and neurophysiological effects of CES in a patient suffering from chronic stress and sleep disturbance. By integrating subjective clinical outcomes with objective electrophysiological data, this report seeks to provide a more detailed understanding of the changes associated with CES treatment and to generate hypotheses for future, larger-scale investigations into its mechanisms of action.

## 2 Case description

### 2.1 Case presentation

**Figure 1:**
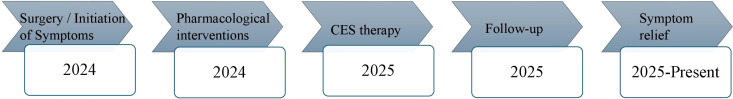
Timeline of the clinical course and interventions for the patient treated with CES.

The patient was a 51-year-old woman who presented with chronic stress-related symptoms and persistent sleep disturbance that had significantly worsened following parathyroid and thyroid surgeries (**Figure 1**). Her clinical history included long-standing sleep disturbances, high physiological stress reactivity, and reduced daytime functioning. Prior pharmacological interventions yielded limited and unsuccessful symptom relief. No prior family history of any psychiatric disease was reported. A structured clinical assessment confirmed diagnoses of chronic stress. Screening for attention-deficit/hyperactivity disorder using the Adult ADHD Self-Report Scale (ASRS) was negative, while no other neurological disorders were identified.

### 2.2 Intervention

The patient participated in a 16-week neuromodulation program of daily CES using the Alpha-Stim AID device (Electromedical Products International, USA). The stimulation waveform consisted of a modified square-wave bipolar current with frequency 0.5 Hz. Current intensity was individually titrated and ranged from 100 to 500 micro-amperes, adjusted to remain below the sensory threshold. Each session lasted 20 minutes and was conducted in a relaxed position. Sessions were performed once daily, for 7 times per week for a total duration of 16 weeks. The stimulation parameters were selected based on prior CES literature (3) and manufacturer guidelines for the Alpha-Stim device, which commonly employ low-frequency (0.5 Hz) microcurrent stimulation within the 100-500 μA range for anxiety and insomnia-related indications. These parameters were consistent with previous clinical studies reporting anxiolytic effects and favorable tolerability profiles (3).

### 2.3 Outcome measures

Clinical and functional outcomes were assessed at baseline and at a 16-week follow-up time point using validated self-report instruments. Health-related quality of life was evaluated with the EuroQoL 5-Dimension 5-Level (EQ-5D-5L). Psychological symptom burden was assessed using the Depression, Anxiety, and Stress Scale (DASS-21) and the Generalized Anxiety Disorder 7-item scale (GAD-7). Subjective sleep quality and sleep-related impairment were measured using the Pittsburgh Sleep Quality Index (PSQI).

### 2.4 Neurophysiological assessment

Resting-state electroencephalography (EEG) was recorded at baseline and after 16 weeks of intervention using the Enobio 20 wireless EEG system (Neuroelectrics, Barcelona, Spain), a CE-marked EEG platform used in clinical and research settings. The system provides 20-channel scalp EEG acquisition with 24-bit signal resolution and a sampling rate of 500 Hz, allowing stable recording of spontaneous cortical activity.

Electrode placement followed the International 10–20 system using a standardized scalp montage covering frontal, central, temporal, parietal, and occipital derivations, with dedicated reference and ground electrodes positioned according to manufacturer specifications. Wet Ag/AgCl electrodes were used to ensure adequate scalp conductivity, and electrode impedances were maintained within clinically acceptable limits prior to recording. Recordings were obtained in a quiet room under standardized ambient conditions with the participant seated comfortably and instructed to remain relaxed while minimizing eye and body movements. Both eyes-open and eyes-closed resting-state conditions were acquired to assess spontaneous background activity, posterior dominant alpha rhythm, and vigilance stability.

Continuous EEG signals were acquired using the Neuroelectrics NIC2 recording platform and visually monitored for signal quality. Recordings were subsequently reviewed offline by an experienced clinical neurophysiologist using longitudinal bipolar, transverse bipolar, and common average reference montages. Recordings were visually inspected for movement, ocular, muscular, and electrode-related artifacts, and artifact-contaminated segments were excluded from interpretation. Descriptive comparative evaluation between baseline and follow-up recordings focused on the organization and stability of the posterior dominant alpha rhythm, the regional prominence of posterior alpha activity, overall hemispheric symmetry, and the presence or absence of focal or epileptiform abnormalities.

### 2.5 Treatment adherence

Treatment adherence was monitored through patient self-report and device usage consistency during scheduled clinical follow-ups. Sessions were performed in a standardized, relaxed setting, with initial instructions and periodic supervision provided by medical staff.

## 3 Results

Following 16 weeks of CES using the Alpha-Stim device, the patient demonstrated clinically meaningful improvements across psychological, quality-of-life, and neurophysiological domains (**Table 1**). No adverse events or treatment discontinuations were reported during the intervention period.

**Table 1:** Changes in clinical outcome measures following 16 weeks of CES.

Measure	Baseline	16-week follow-up	% change
DASS-21 Depression	12	4	− 66.7
DASS-21 Anxiety	12	0	− 100
DASS-21 Stress	32	8	− 75
GAD-7	17	2	− 88.2
EQ-5D-5L General Health	60	75	+ 25
EQ-5D-5L Role Emotional	62.5	82.5	+ 32
EQ-5D-5L Mental Health	37.5	72.5	+ 93.3
PSQI Total Score	12	11	− 8.3

Negative percentage values indicate improvement (symptom reduction) for the DASS-21 and GAD-7 measures, where lower scores reflect reduced psychological distress. Positive percentage values indicate improvement for EQ-5D-5L subscales, where higher scores represent better perceived health and functioning. For the PSQI, negative values indicate improvement, as lower scores correspond to better sleep quality.

### 3.1 Assessment findings

**Figure 2:**
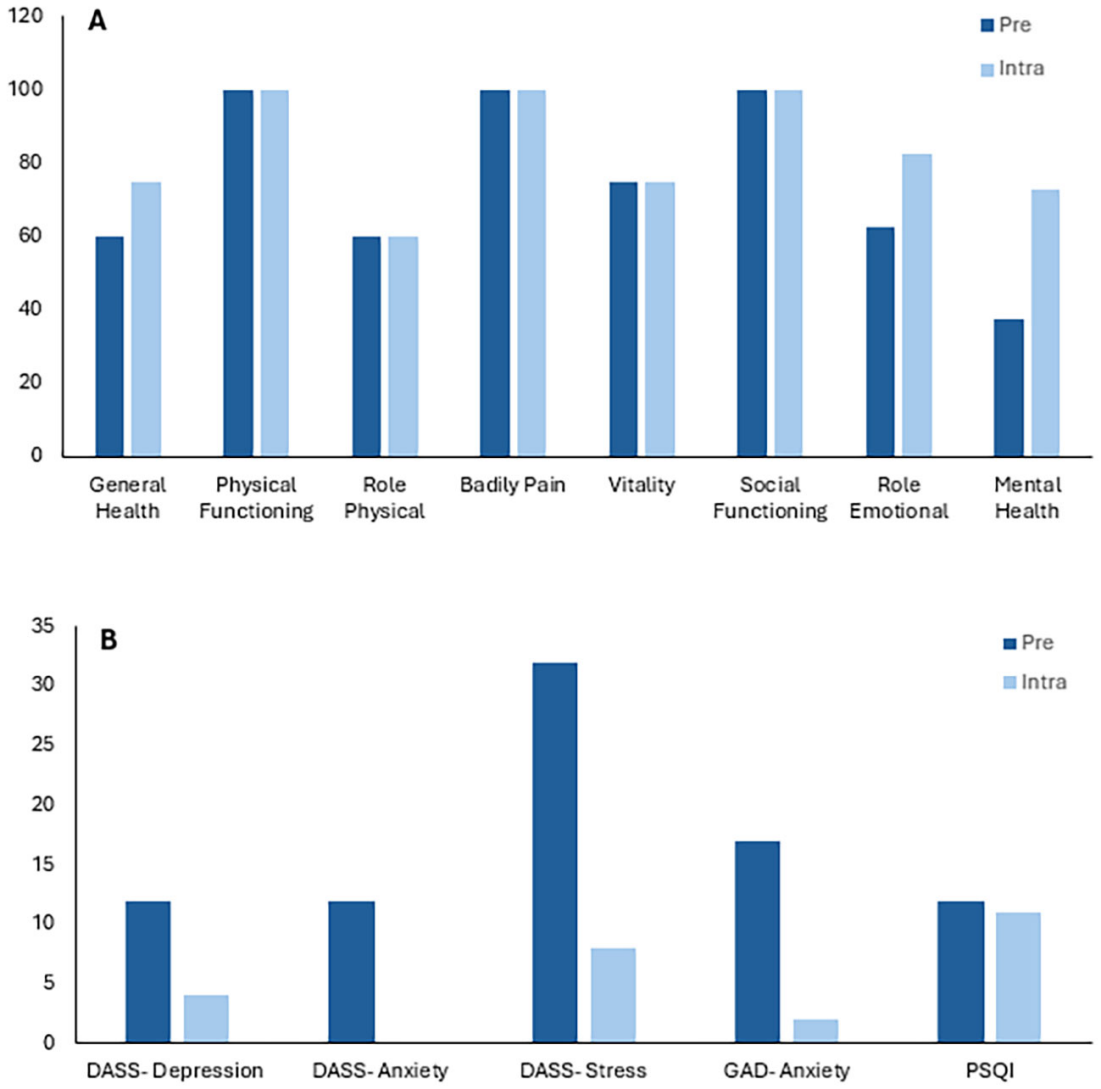
Pre- and post-CES treatment scores on **(A)** EQ-5D-5L and **(B)** DASS-21, GAD-7, and PSQI scales.

Health-related quality of life, assessed using the EQ-5D-5L, showed notable improvement (**Figure 2A**), particularly in mental health-related domains. The general health subscale increased from 60 to 75, role emotional functioning improved from 62.5 to 82.5, and the mental health component score rose from 37.5 to 72.5. Physical functioning and social functioning domains remained consistently high across assessments, indicating stable physical health status and preserved social engagement throughout the observation period. Marked reductions in psychological distress were also observed across all DASS-21 subscales (**Figure 2B**). Depressive symptoms decreased from 12 (mild range) at baseline to 4 (within normal limits) at follow-up. Anxiety scores declined from 12 (moderate range) to 0 (normal), while stress scores were reduced from 32 (severe range) to 8 (normal). These changes indicate a substantial improvement in affective regulation and stress responsiveness. Consistent with these findings, anxiety severity measured by the GAD-7 (**Figure 2B**) decreased from 17 (severe) at baseline to 2 (normal range) at follow-up, further supporting a robust reduction in clinically relevant anxiety symptoms. Subjective sleep quality, as measured by the Pittsburgh Sleep Quality Index (PSQI), demonstrated a modest improvement (**Figure 2B**), with total scores decreasing from 12 to 11. While the patient continued to report sleep-related difficulties, this change suggests a slight reduction in overall sleep disturbance during the initial treatment phase.

### 3.2 Neurophysiological findings

**Figure 3:**
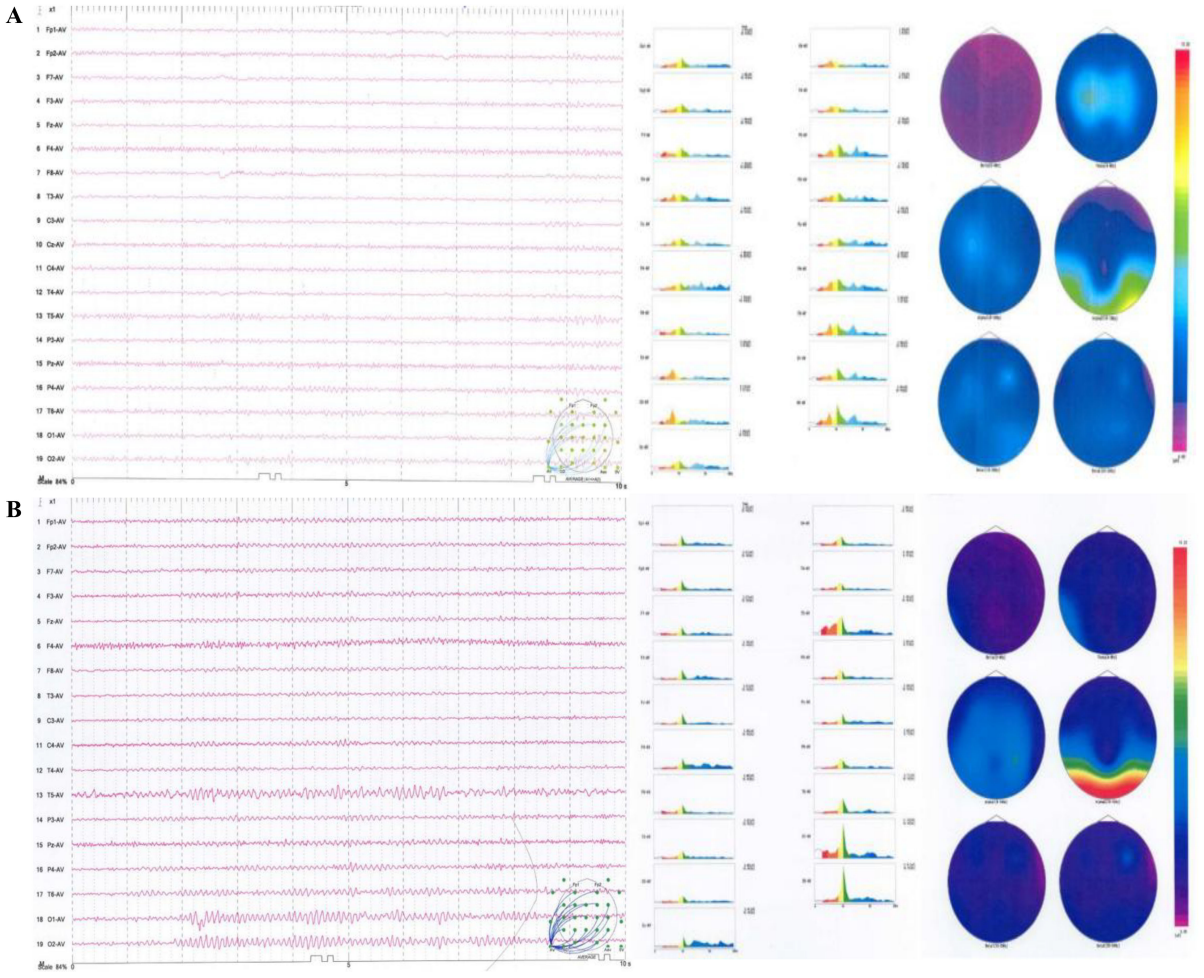
EEG changes before **(A)** and after **(B)** CES therapy using the average EEG montage.

Baseline resting-state EEG demonstrated a preserved but relatively weak posterior dominant rhythm within the alpha frequency range, with low-to-moderate amplitude and limited posterior spatial concentration during relaxed wakefulness (**Figure 3A**). The background activity appeared less distinctly organized, with mild asymmetrical distribution of anterior alpha activity across homologous frontal derivations.

Following 16 weeks of daily CES intervention (**Figure 3B**), repeat EEG recordings demonstrated a more clearly organized posterior dominant alpha rhythm with greater prominence over occipital and parietal regions. Posterior alpha activity appeared more stable and spatially concentrated during the eyes-closed resting condition. In addition, frontal hemispheric distribution appeared more balanced at follow-up, with reduced asymmetrical variability across anterior homologous channels compared to baseline. No epileptiform discharges, focal slowing, or other clinically significant abnormalities were identified at either assessment point. Overall, these descriptive electrophysiological findings are consistent with improved resting-state cortical organization, increased posterior alpha activity, and reduced frontal asymmetry following the intervention, although causal inferences cannot be established.

## 4 Discussion

This case report describes clinically meaningful psychological, autonomic, and neurophysiological changes following 16 weeks of daily CES in a patient with chronic stress and pharmaco-resistant sleep disturbance. The convergence of improvements across validated assessment measures and objective EEG markers supports the clinical plausibility of CES as a neuromodulatory intervention in stress-related neuropsychiatric presentations.

The most pronounced clinical effects were observed in anxiety, stress, and depressive symptoms, with DASS-21 and GAD-7 scores improving from moderate-to-severe ranges to within normative limits. This magnitude of change exceeds commonly reported minimal clinically important differences and is consistent with prior controlled and meta-analytic studies demonstrating anxiolytic effects of CES, particularly in stress- and anxiety-related conditions (8, 9). Notably, anxiety and stress showed the greatest reductions, suggesting that CES may exert stronger effects on hyperarousal and autonomic dysregulation than on core depressive symptomatology. This pattern is clinically relevant given the patient’s limited responsiveness to pharmacological interventions, a context in which CES has been proposed as a viable adjunct or alternative, particularly when medication efficacy or tolerability is suboptimal (10). Sleep-related outcomes demonstrated only modest improvement at the mid-treatment time point, with PSQI scores showing minimal change that did not meet commonly accepted thresholds for clinically meaningful improvement. This finding does not necessarily indicate limited efficacy but may reflect the indirect mechanism through which CES influences sleep. In chronic stress states, insomnia is often maintained by cognitive and autonomic hyperarousal rather than primary sleep drive dysfunction. By reducing pre-sleep anxiety and physiological arousal, CES may contribute to gradual improvements in sleep over longer treatment periods or when combined with behavioral sleep interventions. This interpretation is consistent with prior studies suggesting that CES may improve sleep primarily through anxiolytic pathways rather than direct hypnotic effects (6, 11). Additionally, the modest change observed may reflect limitations in the sensitivity of the PSQI, as well as the persistence of underlying physiological or endocrine factors contributing to sleep disturbance.

Neurophysiological findings provide objective evidence consistent with the observed clinical changes. Post-treatment EEG demonstrated increased posterior alpha power, improved alpha-band definition, and resolution of frontal asymmetry. Alpha oscillations are widely regarded as markers of cortical inhibition, sensory gating, and emotional regulation, with reduced alpha activity commonly observed in states of chronic stress and anxiety (12, 13). Increased posterior alpha power has been associated with reduced limbic hyperactivity and improved stress resilience, while frontal alpha asymmetry has been linked to affective dysregulation and anxiety vulnerability. The reduction in frontal asymmetry observed in this case may reflect improved anterior cortical regulation and enhanced top-down control over threat-related processing.

From a mechanistic perspective, CES is hypothesized to influence thalamocortical and limbic-hypothalamic circuits involved in arousal regulation, emotional processing, and autonomic balance (3, 6). Indirect modulation of neurotransmitter systems, including serotonergic and noradrenergic pathways, as well as downstream effects on hypothalamic–pituitary–adrenal (HPA) axis activity, have also been proposed (6, 12). In patients with endocrine vulnerability, such as those with thyroid or parathyroid dysfunction, these regulatory effects may be particularly clinically relevant, given the bidirectional interactions between stress physiology and endocrine function. While causal mechanisms cannot be established from a single case, the alignment between subjective improvement and EEG normalization is consistent with a potential neuromodulatory effect, although alternative explanations such as placebo effects or natural variability cannot be excluded.

The patient’s history of thyroid and parathyroid surgery is clinically relevant, as endocrine dysregulation is closely linked to stress physiology, autonomic balance, and sleep regulation. Thyroid hormone alterations may influence CNS excitability, while parathyroid-related changes in calcium homeostasis may affect neuronal signaling. These factors may have contributed to symptom persistence and may interact with neuromodulatory effects of CES.

This case underscores the importance of integrating CES within a multidisciplinary neurorehabilitation framework. Rather than functioning as a standalone intervention, CES may be most effective when embedded within comprehensive care models that include clinical monitoring, psychoeducation, and lifestyle support. Such integration aligns with emerging perspectives emphasizing individualized neuromodulation approaches tailored to patient-specific neurophysiological and psychosocial profiles. However, this study has several limitations. As a single-case report, findings cannot be generalized and causal inference is not possible. Clinical outcomes relied primarily on self-report measures, without clinician-rated or objective behavioral assessments. No objective sleep measures (e.g., actigraphy or polysomnography) were included, limiting the interpretation of sleep-related changes. EEG analysis was descriptive and based on clinical visual assessment without quantitative statistical comparisons. Moreover, the absence of sham control conditions precludes definitive exclusion of placebo effects.

Additionally, the absence of intermediate time points limits the ability to characterize the trajectory of clinical and neurophysiological changes over time. EEG findings remain correlational and do not establish specific mechanistic pathways. Longer-term follow-up and replication in larger, controlled cohorts are required to assess the durability, specificity, and generalizability of the observed effects. Overall, findings should be interpreted as associative rather than causal.

In summary, this case report suggests that daily CES was associated with improvements in psychological distress, quality of life, and EEG markers associated with cortical regulation in a patient with chronic stress and treatment-resistant symptoms. While sleep improvements were modest at the mid-treatment assessment, the overall pattern of findings supports CES as a promising adjunctive intervention for complex neuropsychiatric and stress-related conditions. Further systematic investigation is warranted to refine patient selection, optimize treatment parameters, and clarify long-term outcomes, while future studies incorporating controlled designs and quantitative EEG metrics are needed to further validate these findings.

### 4.1 Patients perspective

“Before CES treatment, I struggled with ongoing stress, anxiety, and poor sleep that did not improve with medication. Over time, I felt calmer, less anxious, and more emotionally stable, with fewer racing thoughts at night. The treatment was easy to tolerate and helped me better manage my daily symptoms.”

## Data Availability

The raw data supporting the conclusions of this article will be made available by the authors, without undue reservation.

## References

[1] Zhang J . The impact of stress on sleep quality: a mediation analysis based on longitudinal data. Frontiers in Psychology (2024). pp. 1–13. doi: 10.3389/fpsyg.2024.1431234. PMC11532129 39498330

[2] Xue Y Wang WD Liu YJ Wang J Walters AS . Sleep disturbances in generalized anxiety disorder: the central role of insomnia. Sleep Med. (2025) 132:106545. doi: 10.1016/j.sleep.2025.106545. PMID: 40318600

[3] Brunye TT Patterson EJ Wooten T Hussey EK . A critical review of cranial electrotherapy stimulation for neuromodulation in clinical and. (2021) 15:1–16. doi: 10.3389/fnhum.2021.625321 PMC7882621 33597854

[4] Shekelle PG Cook IA Miake-Lye IM Booth MS Beroes JM Mak S . Benefits and harms of cranial electrical stimulation for chronic painful conditions, depression, anxiety, and insomnia: a systematic review. Ann Intern Med. (2018) 168:414–21. doi: 10.7326/M17-1970. PMID: 29435567

[5] Guleyupoglu B Schestatsky P Edwards D Fregni F Bikson M . Classification of methods in transcranial electrical stimulation (tES) and evolving strategy from historical approaches to contemporary innovations. J Neurosci Methods. (2013) 219:297–311. doi: 10.1016/j.jneumeth.2013.07.016. PMID: 23954780 PMC3833074

[6] Kirsch DL Nichols F . Cranial electrotherapy stimulation for treatment of anxiety, depression, and insomnia. Psychiatr Clin North Am. (2013) 36:169–76. doi: 10.1016/j.psc.2013.01.006. PMID: 23538086

[7] Kirsch D Smith R . The use of cranial electrotherapy stimulation in the management of chronic pain: a review. NeuroRehabilitation. (2000) 14:85–94. doi: 10.3233/NRE-2000-14204 11455071

[8] Ching PY Hsu TW Chen GW Pan CC Chu CS Chou PH . Efficacy and tolerability of cranial electrotherapy stimulation in the treatment of anxiety: a systemic review and meta-analysis. Front Psychiatry. (2022) 13:899040. doi: 10.3389/fpsyt.2022.899040. PMID: 35757229 PMC9218324

[9] Liu SY Chen R Wang CH Banda KJ Sung CM Chang LF . Efficacy of cranial electrotherapy stimulation for treating primary and secondary depression in adults: a meta-analysis of randomized controlled trials. J Affect Disord. (2025) 382:488–97. doi: 10.1016/j.jad.2025.04.074. PMID: 40286924

[10] Barclay TH Barclay RD . A clinical trial of cranial electrotherapy stimulation for anxiety and comorbid depression. J Affect Disord. (2014) 164:171–7. doi: 10.1016/j.jad.2014.04.029. PMID: 24856571

[11] Okano K Lee MM Hart-Pomerantz H Smith M Sandone MK Harvey T . Effects of repeated cranial electrotherapy stimulation on physiological and behavioral responses to acute stress: a double-blind randomized clinical trial. Front Hum Neurosci. (2025) 19:1641801. doi: 10.3389/fnhum.2025.1641801. PMID: 40881935 PMC12380913

[12] Feusner JD Madsen S Moody TD Bohon C Hembacher E Bookheimer YS . Effects of cranial electrotherapy stimulation on resting state brain activity. Brain Behav. (2012) 2:211–20. doi: 10.1002/brb3.45. PMID: 22741094 PMC3381625

[13] Ippolito G Bertaccini R Tarasi L Di Gregorio F Trajkovic J Battaglia S . The role of alpha oscillations among the main neuropsychiatric disorders in the adult and developing human brain: evidence from the last 10 years of research. Biomedicines. (2022) 10. doi: 10.3390/biomedicines10123189. PMID: 36551945 PMC9775381

